# Imaging of head and neck mucosa-associated lymphoid tissue lymphoma (MALToma)

**DOI:** 10.1186/s40644-020-00380-5

**Published:** 2021-01-12

**Authors:** K. W. S. Ko, Kunwar S. Bhatia, Qi Yong H. Ai, Ann D. King

**Affiliations:** 1 Department of Radiology and Imaging, Queen Elizabeth Hospital, 30 Gascoigne Road, Kowloon, Hong Kong, SAR China; 2 Department of Imaging, St Mary’s Hospital, Imperial College Healthcare, National Health Service Trust, London, UK; 3 Department of Imaging and Interventional Radiology, The Chinese University of Hong Kong, Prince of Wales Hospital, 30–32 Ngan Shing Street, Shatin, New Territories, Hong Kong, SAR China

**Keywords:** Mucosa-associated lymphoid tissue, Marginal zone B-cell lymphoma, Imaging, Head and neck

## Abstract

Marginal zone B-cell lymphoma of mucosa-associated lymphoid tissue (MALToma) arises in extranodal sites in the head and neck. Chronic inflammatory, infectious or autoimmune conditions are implicated in its pathogenesis. Within the head and neck, MALToma is often multifocal and indolent and the imaging appearances may be mistaken for non-malignant disease in the head and neck. The aim of this article is to illustrate the varied radiological and clinical features of MALToma in the head and neck, an awareness of which is needed for timely and correct diagnosis to guide subsequent disease management.

## Background

MALToma is an extranodal B-cell non-Hodgkin lymphoma (NHL) and although it accounts for only7–9% of NHLs in the head and neck the incidence is on the rise [1, 2]. MALToma presents at mean age of 52–59 years but affects a wide age range of 21–92 years [1, 3] and unlike other head and neck NHLs is more prevalent in females [3, 4]. MALToma also differs from most other B-cell NHLs in that it has a predilection for salivary, thyroid and lacrimal glandular tissues and the orbits [1, 3, 4]. Chronic infection, inflammation and autoimmune disease lead to abnormal lymphoid proliferation predisposing to MALToma which arises within marginal zone B lymphocytes [5] and is often multifocal.

The indolent course of MALToma hinders diagnosis, especially in sites of pre-existing chronic disease. Distinction of an inflammatory process from MALToma is also challenging on fine needle aspiration cytology and image guided core biopsy is frequently used to balance diagnostic efficacy against the risk of an invasive open biopsy [6, 7]. The optimal treatment approach for MALToma is controversial. Early-stage disease is treated by radiotherapy alone [8–11] or for cases with MALToma of salivary and thyroid glands, by surgical resection, with or without adjuvant radiotherapy/chemotherapy [11]. Advanced-stage disease is treated by a combination of radiotherapy and chemotherapy [11–14]. Other notable therapies are immunotherapy agents, which also alleviate background Sjögren’s disease [15] and antibiotics for Chlamydia-associated orbital MALToma [16]. MALToma is difficult to cure and relapse is common, but 5-year overall survival is high (85–96%) for locally indolent disease [11]. Transformation to high grade lymphoma and nodal / disseminated disease carry a worse prognosis [1].

## General imaging features

### Primary site

On all imaging modalities MALToma produces solitary or multiple solid nodules which are usually homogeneous without cysts, necrosis or calcification (Figs. 1, 2, 3, 4, 5, 6, 7, 8). Tumour margins are usually well-defined but can be poorly-defined [7, 17, 18].

**Fig. 1:**
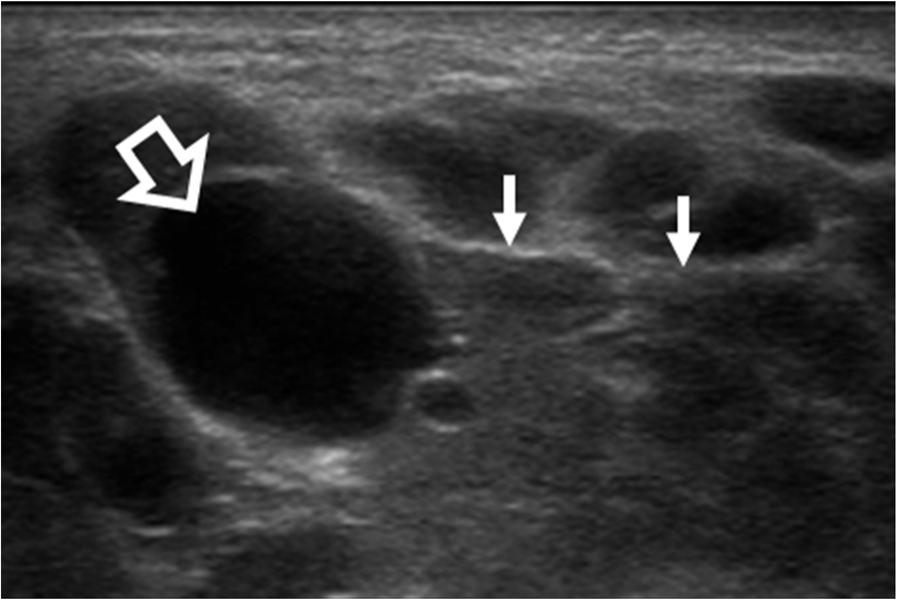
Ultrasound of a 33-year-old woman with parotid MALToma presenting as bilateral parotid gland swelling for 7 years. The parotid gland shows a dominant homogeneous very hypoechoic solid nodule that could be mistaken for a cyst (open arrow) against a background of numerous small homogeneous solid hypoechoic nodules interspersed with echogenic strands (arrows)

**Fig. 2:**
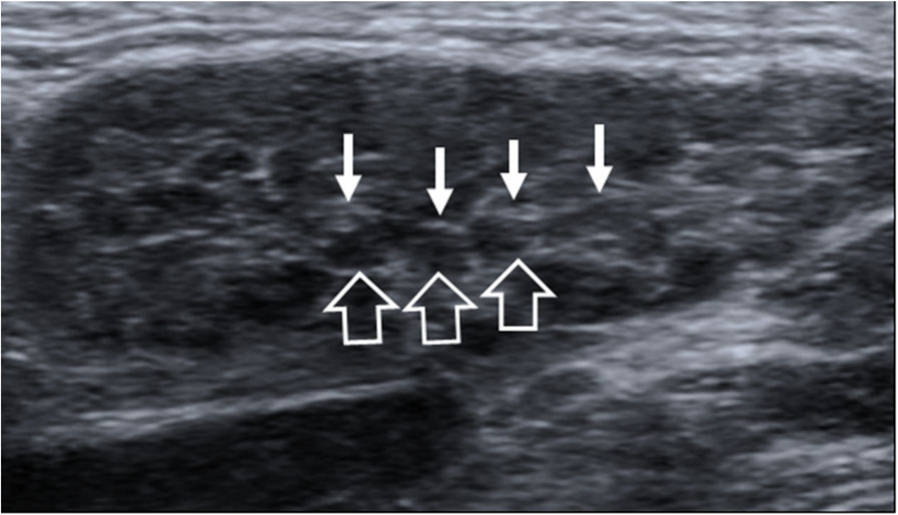
Ultrasound of a 72-year-old man with right submandibular MALToma presenting as bilateral parotid and submandibular gland swelling for 6 months. The right submandibular gland shows replacement by multiple small hypoechoic nodules of similar size (open arrows) separated by echogenic strands (arrows). The appearance overlaps with that of Sjögren’s syndrome without a dominant nodule to indicate this was MALToma

**Fig. 3:**
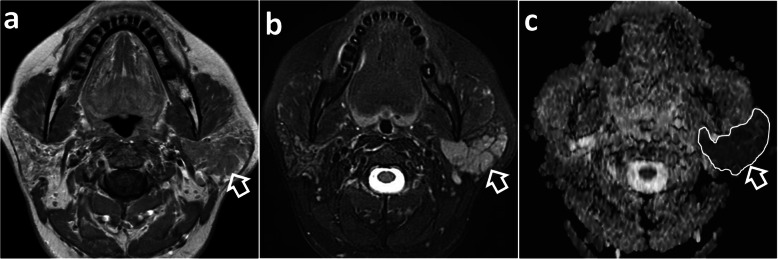
Magnetic resonance imaging of a 40-year-old woman with Sjögren’s disease and left parotid MALToma presenting with long-standing bilateral parotid swelling and 2 years increasing swelling on the left side. **a** Axial T1-weighted post-contrast image shows a moderately enhancing, homogenous lobulated left parotid mass (open arrow). Both parotid glands exhibit background changes of Sjögren’s disease. **b** Axial T2-weighted image with fat saturation shows a left parotid mass with intermediate signal intensity (open arrow). Both parotid glands exhibit background changes of Sjögren’s disease. **c** Axial apparent diffusion coefficient (ADC) map shows markedly restricted diffusion (open arrow) with a very low ADC value of 0.64 × 10^− 3^ mm^2^/s (white contour)

**Fig. 4:**
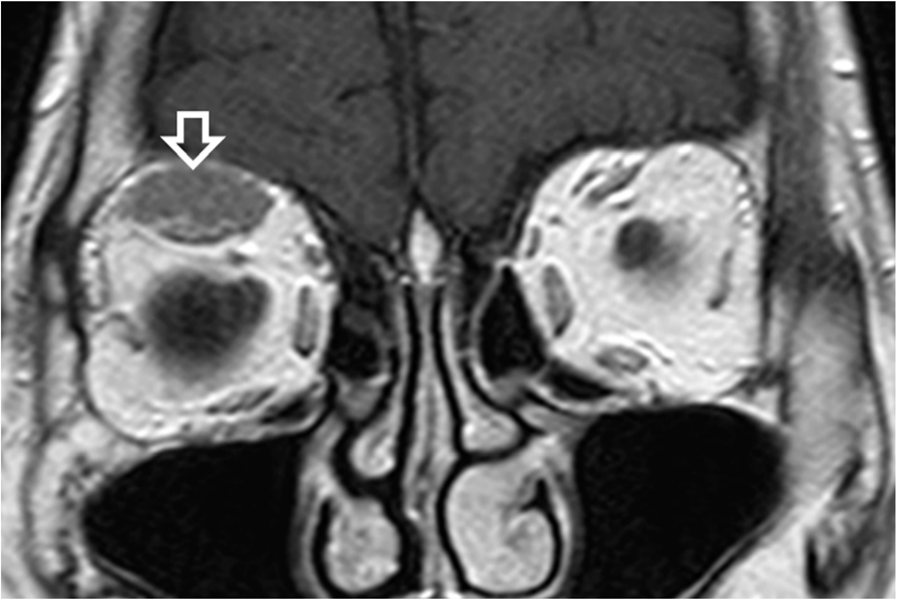
Magnetic resonance imaging of an 82-year-old man with right orbital MALToma presenting as painless orbital swelling. Coronal T1-weighted post-contrast image shows a homogeneous moderately contrast-enhancing MALToma in the right superior rectus muscle (open arrow), a common site of orbital MALToma

**Fig. 5:**
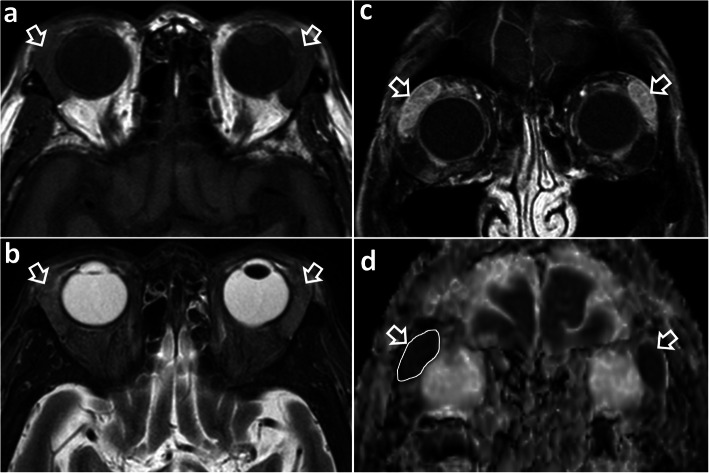
Magnetic resonance imaging of an 87-year-old woman with insidious onset of bilateral eyelid swelling and proptosis for 2 years, who had bilateral lacrimal gland MALToma. **a** Axial T1-weighted image shows bilateral enlargement of the lacrimal glands with homogeneous intermediate signal intensity (open arrows). **b** Axial T2-weighted image with fat saturation shows bilateral enlargement of the lacrimal glands with homogeneous intermediate signal intensity (open arrows). **c** Coronal T1-weighted post-contrast image with fat saturation shows bilateral enlargement of the lacrimal glands (open arrows) with moderate homogeneous contrast enhancement. **d** Coronal apparent diffusion coefficient (ADC) map shows bilateral enlargement of the lacrimal glands with markedly restricted diffusion (open arrows) with a very low ADC value of 0.56 × 10^− 3^ mm^2^/s (white contour)

**Fig. 6:**
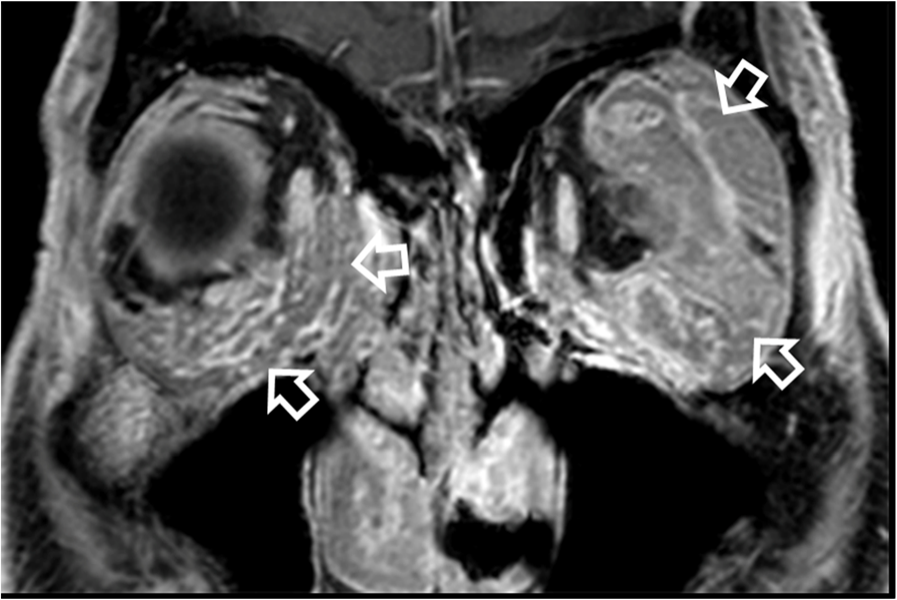
Magnetic resonance imaging of a 72-year-old man with bilateral orbital MALToma presenting as orbital swelling. A coronal T1-weighted post-contrast image with fat saturation reveals extensive bilateral extraconal and intraconal orbital involvement and involvement of the eyelids (open arrows)

**Fig. 7:**
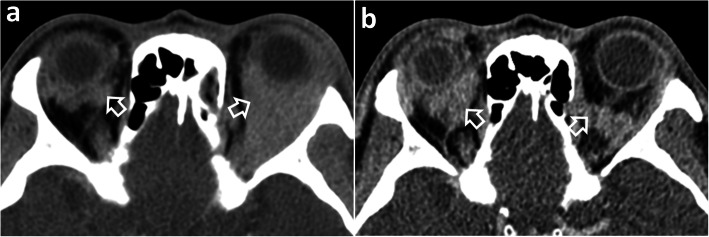
Computed tomography of a 48-year-old man with a relapse of orbital MALToma illustrating the indolent nature of this disease. **a** Axial contrast-enhanced image at the initial presentation when the patient complained of left-sided proptosis and limited ocular movement. There is a diffuse homogeneous intraconal and extraconal left orbital MALToma (open arrow), with a small right intraconal MALToma (open arrow). The patient responded to radiotherapy. **b** Axial contrast-enhanced image 8 years later when the patient developed of right-sided proptosis. There is relapse in the right orbit (open arrow) and a clinically occult left orbital relapse (open arrow)

**Fig. 8:**
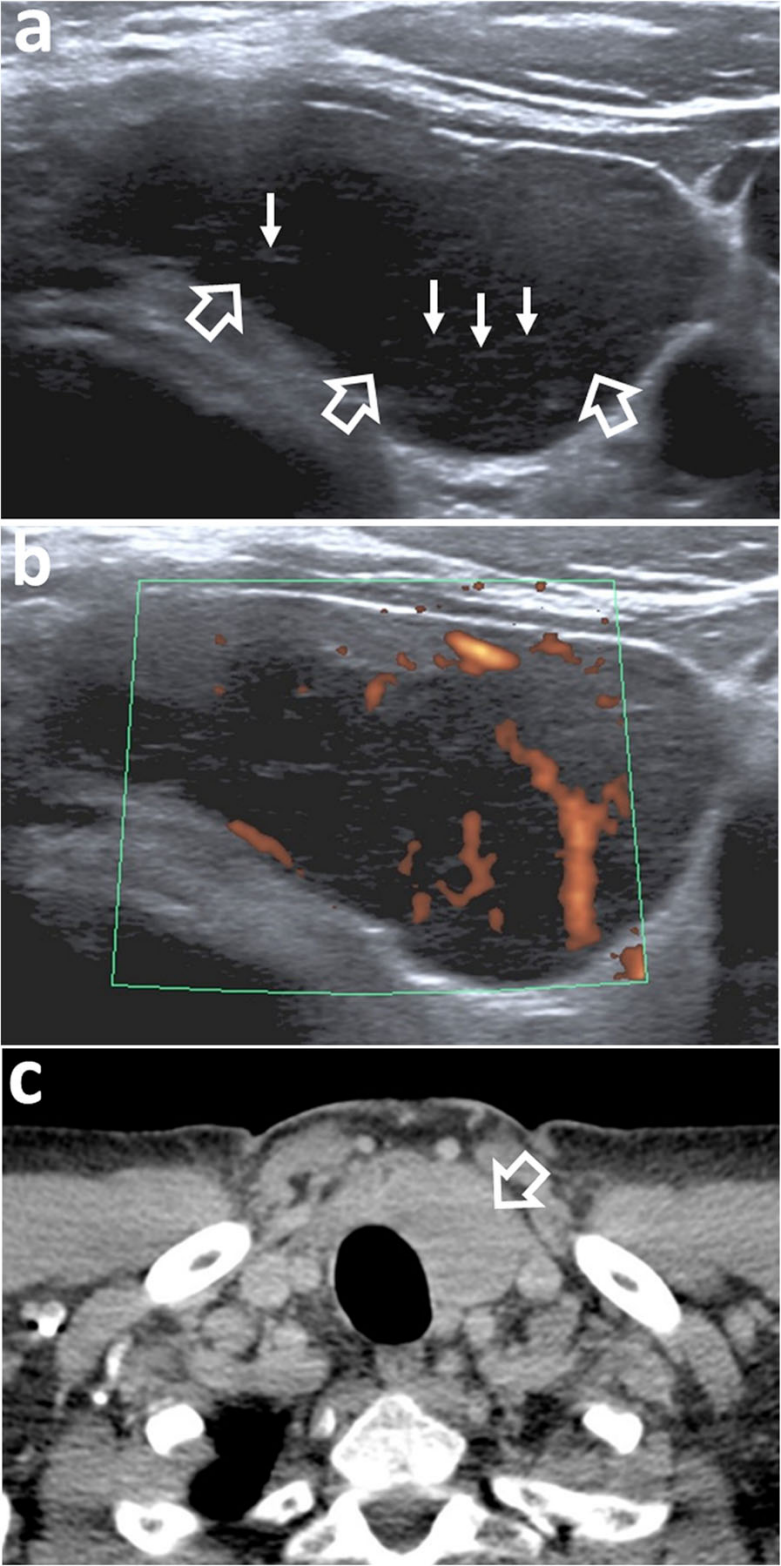
Ultrasound (US) and computed tomography (CT) in a 58-year-old man with left thyroid lobe MALToma, presenting with neck swelling. **a** US shows the left lobe is enlarged and occupied by a lobulated, markedly hypoechoic mass (open arrows) with echogenic fibrous strands (arrows). There is sparing of the normal thyroid parenchyma anterior to the tumour. **b** Colour Doppler US shows internal vascularity. **c** Axial contrast-enhanced CT image shows a solitary mildly enhancing nodule in the left thyroid lobe (open arrow)

Ultrasound (US) is often the first investigation because of the superficial site of the glandular tissues in the head and neck. MALToma is markedly hypoechoic (Figs. 1, 2, and 8a), may contain fibrous striations comprising fine linear echogenic strands or wider denser echogenic bands [17–20] (Figs. 1, 2, and 8a) and shows internal vascularity on colour Doppler [7] (Fig. 8b). Internal striations and vascularity prevent misinterpreting a markedly hypoechoic MALToma for a cyst. Posterior acoustic enhancement is described [17–19] but is less frequently observed following advances in post-processing US technology [18, 21]. US also guides the site of biopsy.

Computed tomography (CT) and magnetic resonance imaging (MRI) are complementary to US and provide comprehensive evaluation of the whole head and neck region, encompassing all lymphatic, extra-lymphatic and nodal sites. MALToma is isoattenuating to mildly hyperattenuating [22], with mild to moderate contrast enhancement on CT [23, 24] (Figs. 7 and 8c). MRI provides better soft tissue contrast than CT, allowing depiction of subtle tumours, especially in a background of pre-existing chronic conditions that predispose to MALToma. The signal intensity of MALToma on MRI is low-intermediate on T1-weighted images, intermediate-mildly high on T2-weighted images, very low on apparent diffusion coefficient (ADC) maps and shows moderate contrast enhancement (Figs. 3, 4, 5, and 6). The ADC maps from diffusion weighted imaging (DWI) are especially useful in lesion characterisation because head and neck lymphoma, typically shows greater restriction of diffusion than most other cancers [25] or inflammatory processes [26] with an average ADC value of 0.65 × 10^− 3^ mm^2^/s, although much lower values are reported [27, 28]. Dynamic contrast-enhanced MRI or dual-phase contrast-enhanced CT can be helpful in lesion characterisation as MALToma exhibits earlier wash-in and higher relative washout than benign tumours or inflammatory processes such as pleomorphic adenoma [29] or IgG4-related disease [30, 31]. Hydrogen-proton MR spectroscopy (MRS) is deemed less useful because similar degrees of choline elevation are found in MALToma and benign lymphoepithelial lesions [32].

The role of 18F-fluorodeoxyglucose positron emission tomography (FDG-PET) in MALToma is controversial. Early reports suggest low detection rates due to low FDG uptake of early-stage MALToma [33]. However, in a recent meta-analysis the overall detection rate by FDG-PET-CT was 71%, being especially high in head and neck (90%) [34].

### Nodal sites

**Fig. 9:**
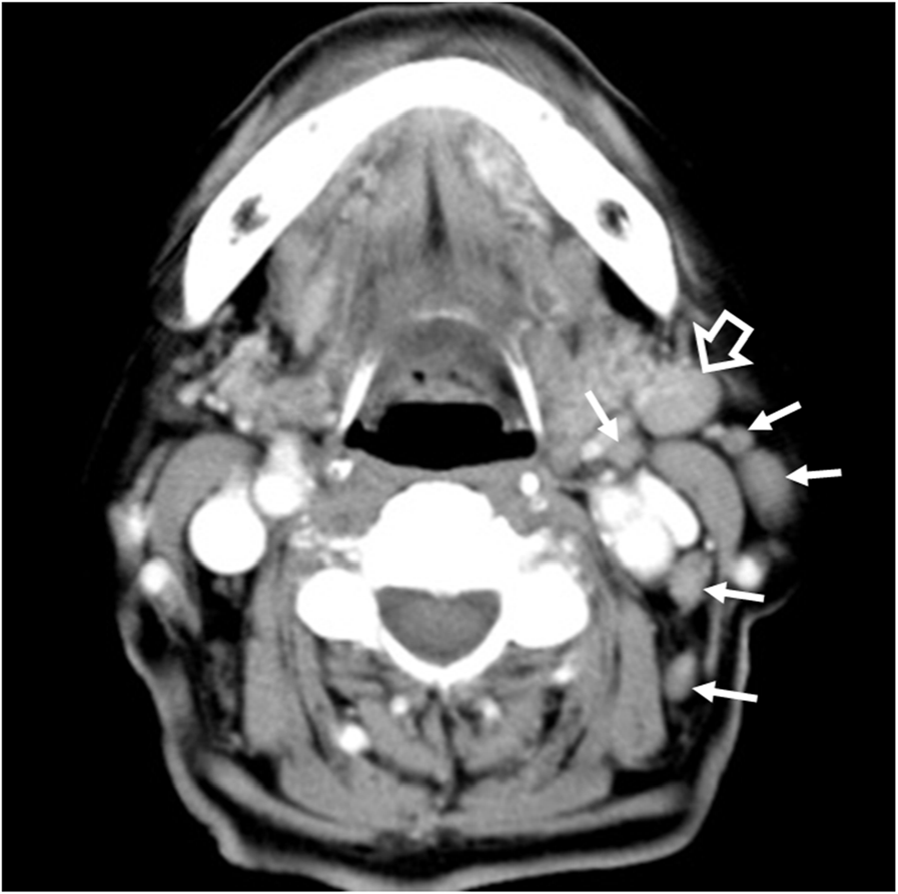
Computed tomography of a 77-year-old woman with left parotid MALToma and nodal disease. Axial contrast-enhanced image shows a slightly enlarged reactive appearing node (open arrow) and a cluster of small non-enlarged nodes (arrows) in the left upper internal jugular chain

Nodal disease occurs in 21% of patients with non-gastric lymphoma [3]. In the head and neck, enlarged nodes with typical lymphomatous features on imaging (round shape, loss or eccentric displacement of fatty hilum, and micronodular pattern on US [21]) are uncommon, although linear echogenic strands within hypoechoic nodes have been observed in MALToma [20]. On the other hand, numerous small nodes are frequently encountered, and these may have reactive features [35], making them difficult to diagnose on any imaging modality (Fig. 9).

## Site-specific clinical and imaging features

### Salivary gland

#### Clinical features

The salivary glands are the most common site of MALToma in the head and neck (46.5 to 81%). The parotid gland accounts for 80% of these MALTomas but as lymphoma accounts for less than 4% of parotid tumours it may be overlooked leading to unnecessary parotidectomy [36]. Patients present with localized swelling, facial nerve palsy is uncommon (4–15%) [37]. Sjögren’s disease is the major predisposing factor for MALToma; 20% of patients have underlying Sjögren’s disease [38] which increases the risk of MALToma by 44 times with a cumulative risk of 6% per year [39]. Less common predisposing conditions are systemic lupus erythematosus, rheumatoid arthritis and IgG4-related disease [40], which can manifest as chronic sclerosing sialoadenitis in the submandibular gland [41].

#### Imaging features

Salivary gland MALToma presents as a solitary solid nodule/ mass in 35% of patients (Fig. 3) with imaging features as described above. Multifocal disease is common, seen in 65% of patients, and can involve single, bilateral or multiple salivary glands, as well as the lacrimal glands [42]. On US, solid nodules can be markedly hypoechoic and mistaken for cysts (Fig. 1). MALToma may also manifest as multiple small hypoechoic solid nodules interspersed with linear echogenic septations of variable thickness which gives rise to a “tortoise-shell” pattern [43] (Figs. 1 and 2). Fibrous echogenic strands are described as fine lines within a hypoechoic area producing a “linear strand pattern” or wider producing a lobulated segmented pattern [20]. Cysts and calcification have been reported in parotid MALToma; but, these features are difficult to separate from the underlying autoimmune/chronic inflammatory disease, which produces lymphoepithelial lesions that manifest as heterogeneous nodules, cysts (lymphoepithelial or secondary to obstructed ducts) and foci of calcification [38].

#### Differential diagnoses of salivary MALToma include

1. Salivary gland tumours: an in-depth discussion of the imaging features of benign and malignant salivary neoplasms is beyond the scope of this article. However, MALToma should be considered in cases with a homogeneous solid nodule/mass that shows marked diffusion restriction, multifocal disease and pre-existing chronic disease, especially Sjögren’s disease.
2. Sjögren’s disease: a simplified salivary gland US scoring system has been developed in which the likelihood of Sjögren’s disease is assigned a grade from 0 to 3. The appearance of grade 3, numerous confluent hypoechoic lesions, overlaps with that of MALToma (Fig. 2) [44]. Therefore, in a gland with pre-existing Sjögren’s disease, a history of recent swelling and presence of dominant homogeneous solid nodule on imaging is important in identifying MALToma and guiding biopsy.

### Orbit

#### Clinical features

The orbit is the second most common site of MALToma in the head and neck [3]. B-cell lymphomas account for 34% of primary orbital malignancies in adults above 60 years of age [30], most of which are MALToma. Common presentations are proptosis or palpable mass with little pain, inflammation or visual impairment [30]. Conjunctival MALToma presents as a vascular fleshy mass over the conjunctiva [45]. Orbital MALToma is indolent but despite initially favourable response to radiotherapy local relapse occurs in 25% of cases at 5 years and 45% at 10 years [46]. Chronic bacterial infections from *Chlamydia psittaci* are reported in up to 89% of cases of ocular adnexal MALToma, hence antibiotics are used for treatment; IgG4-related dacryoadenitis is another predisposing factor [47].

#### Imaging features

Orbital MALToma presents as a solid nodule/ mass with imaging features as described above. It has a predilection for the superolateral quadrant of orbit where it involves the lacrimal gland and/or superior rectus muscle (Figs. 4, 5, and 6). Other sites include other extraocular muscles (especially lateral rectus muscle), conjunctiva, eyelids and extraconal and intraconal soft tissues (Fig. 6) [48]. MALToma may be localised to one site or may spread to involve multiple adjacent sites, with bilateral disease (Fig. 5) in 15% of patients at presentation [30] (Figs. 5, 6, and 7). Bone erosion and hyperostosis are uncommon. Intraocular extension is also uncommon and suggests the rare possibility of intraocular uveal MALToma, which spreads outside the globe via the optic nerve [48]. MRI is generally preferred over CT for orbital imaging due to better soft tissue contrast and avoidance of ionising radiation. The indolent nature of MALToma with long periods of quiescence is demonstrated in Fig. 7.

#### Differential diagnoses of orbital MALToma include

1. IgG4-related orbital pseudo-tumour: higher ADC value (~ 1.40 × 10^− 3^ mm^2^/s) [49] on DWI, lower signal on T2-weighted MRI [49] and hyperenhancement rather than washout on delayed contrast CT [31, 50]
2. Metastasis: higher mean ADC (~ 1.20 × 10^− 3^ mm^2^/s) on DWI [49].
3. Thyroid orbitopathy: more likely to be bilateral, with retro-orbital fat oedema and involvement of multiple extra-ocular muscles (including inferior and medial rectus, which are less commonly affected in MALToma) occurring in a patient with thyroid disease.
4. Lacrimal gland carcinoma: more likely to be heterogeneous with adjacent bone scalloping or punctate calcification [51].

### Thyroid gland

#### Clinical features

The thyroid is the third most common site of MALToma in the head and neck (10% of all cases) [3], often presenting with neck swelling. Although a rapidly enlarging painless goitre or compressive symptoms such as stridor, hoarseness and dysphagia are associated with high-grade B-cell lymphomas, this presentation has been observed in some low-grade MALTomas or in rare cases of transformation to high-grade lymphomas [52]. Systemic dissemination is uncommon. Chronic inflammatory disease is a major predisposing factor [37], especially Hashimoto’s thyroiditis which is found in 92% of patients with thyroid MALToma [37]; Riedel’s thyroiditis is a less common risk factor [53].

#### Imaging features

Thyroid MALToma presents as a solid nodule/mass (Fig. 8) with imaging features as described above. These include markedly hypoechoic areas with internal vascularity and a striated appearance from linear echogenic strands and bands [17–20] (Fig. 8a and b). Partial destruction of these strands has been shown in an area of MALToma which contained more aggressive diffuse large B cell lymphoma components [20]. MALToma presents with variable patterns which are similar to that seen in salivary gland MALToma and include a solitary lesion (Fig. 8), multiple lesions, diffuse pattern with intervening fibrous strands, nodular-segmental pattern or a mixture of these patterns [20, 54]. There is frequently a background of chronic parenchymal disease.

#### Differential diagnoses of thyroid MALToma include

1. Thyroid gland tumours: discussion of the imaging features of benign and malignant thyroid tumours is beyond the scope of this article, but MALToma should be considered when US shows solitary/multiple solid hypoechoic/ markedly hypoechoic homogeneous nodules with no cystic component or calcification, especially when there is a history of hypothyroidism or background parenchymal abnormalities consistent with Hashimoto’s disease.
2. Hashimoto’s disease: imaging appearance of diffuse MALToma may overlap with Hashimoto’s disease although MALToma tends to be of lower density on non-contrast enhanced CT images [19], lower echogenic on US and cause more asymmetrical enlargement of the right and left lobes [17]. Like salivary gland MALToma, a history of recent swelling and evaluation for a dominant homogeneous solid nodule for biopsy are crucial. The value of PET-CT in such cases is controversial, although some reports suggest that thyroid MALTomas are more FDG-avid than the underlying chronic thyroiditis [55, 56].

### Other sites

About 12% of head and neck MALTomas arise at other sites in the head and neck, most commonly along the upper airway in Waldeyer’s ring and the larynx. Again, they are usually indolent but they may present with symptoms of luminal obstruction [57]. MALToma may also arise in minor salivary gland tissues in the head and neck such as in the palate [58]. The imaging features are similar to those described above. The main differential diagnosis of MALToma at these sites is squamous cell carcinoma which tends to show higher ADC values and is less commonly multifocal.

## Conclusion

MALTomas in the head and neck are indolent lymphomas characterised by long periods of quiescence followed by relapse. They occur in extralymphatic sites, most notably the salivary glands, orbits and thyroid gland. They are often multifocal and arise at sites with pre-existing chronic disease (e.g. Sjögren’s disease in the salivary glands, chlamydial infection in the orbits and Hashimoto’s disease in the thyroid). MALTomas commonly manifest radiologically as solitary or multiple solid nodules that are fairly homogeneous without calcification or cysts, showing very low echogenicity, marked restriction of diffusion on DWI and low FDG activity. However, imaging findings may be confounded by presence of chronic disease. In such cases, a thorough search should be made to identify a dominant solid nodule. Awareness of clinical features and radiological appearances of MALToma with high index of suspicion are essential to make a radiological diagnosis of this entity to guide subsequent management.

## Data Availability

Not applicable.

## Abbreviations

ADC: Apparent diffusion coefficient
CT: Computed tomography
DWI: Diffusion weighted imaging
FDG-PET: 18F-fluorodeoxyglucose positron emission tomography
MALToma: Mucosa-Associated Lymphoid Tissue Lymphoma
MRI: Magnetic resonance imaging
NHL: Non-Hodgkin lymphoma
US: Ultrasound
